# Socioeconomic determinants of crop diversity in Bule Hora Woreda, Southern Ethiopia

**DOI:** 10.1016/j.heliyon.2022.e09489

**Published:** 2022-05-21

**Authors:** Belay Maru, Melesse Maryo, Getahun Kassa

**Affiliations:** a Wondo Genet College of Forestry and Natural Resources, Hawassa University, P.O. Box 128, Shashemene, Ethiopia; b Ethiopian Biodiversity Institute, Addis Ababa, Ethiopia; c Bule Hora University, Ethiopia

**Keywords:** Crop diversity, Shannon-wiener index, Socioeconomic

## Abstract

Crop diversification on the farm is a useful approach, especially in developing countries, where agriculture is the primary source of income. Crop diversity management on the farm is critical for reducing poverty, increasing farm revenue, creating jobs, and ensuring long-term agricultural sustainability by maintaining biodiversity, soil, and water resources. Despite their relevance, several variables are currently affecting farmers' crop production decisions. The purpose of this research was to see how socioeconomic factors influence crop diversification. We chose randomly 84 sample household heads from four kebeles to collect socioeconomic and on-farm data. The Shannon-Wiener index (SWI) and crop species richness were used to assess crop diversity. A multiple stepwise linear regression model was used to evaluate the data. Crop diversity was positively and significantly related to household farm size, animal size and composition, annual income, and the location's altitudinal gradient. A lack of road infrastructure and market connections constrained farmers' crop diversification options. It's vital to connect distant areas with road networks and market ties to promote farm-level crop diversification.

## 1 Introduction

Crop diversification is the addition of new crops to existing cropping systems (Adjimoti et al., 2017). Crop diversification is an important agricultural policy, particularly in the development and prosperity of rural areas. Crop diversification significantly contributed to the livelihoods of low-income and small-scale farmers, particularly in developing nations (Abdalla et al., 2013). Crop diversity on farms may be a strategy to boost biodiversity while preserving ecology (Fahrig et al., 2011, 2015). Crop heterogeneity (the presence of several types of crops nearby), polyculture, intercropping, and crop rotation can all be utilized to achieve crop diversity (Thrupp, 2004; Feliciano, 2019).

Crop diversity is important for reducing the negative environmental impacts of agricultural production (Cutforth et al., 2001); providing ecological services such as pollination and pest control (Garibaldi et al., 2013); as well as having a longer growing season (Kasem and Thapa, 2011), which helps to combat climate change by lowering atmospheric CO2 levels. Because each crop has its own set of weather sensitivities, having a diverse crop mix might help farmers avoid a range of climate-related disasters (floods, droughts, hurricanes, etc) (Lin, 2011). In Ethiopia, where this study was conducted, diverse contributions of farm-level crop varieties are common.

Ethiopia is one of the world's richest genetic resource centers in terms of crop diversity, because of its diverse farming practices, socioeconomics, cultures, and agro-ecologies (IBC, 2007). Ethiopian farmers grow a wide range of crops in various sections of the country. However, crop diversification strategies at the farm level are influenced by both environmental and social factors. Several studies on crop diversification among farmers have been conducted in Ethiopia over the last few decades (Edwards, 1991; Gollin and Smale, 1999; Brush, 2004; Sebsebe and Edwards, 2006; Teshome, 2006; Sinafikeh et al., 2009; Tesfaye et al., 2010; Edilegnaw et al., 2011; Tesfaye, 2013; Emana et al., 2015). However, there have been no studies on the difficulty of producing a range of crops among these and other research works on farm-level crop diversification specific to the study location. The purpose of this study was to determine the most common crop varieties and the percentage of land under cultivation, as well as the socioeconomic and physical factors that affect farmers' crop production decisions.

## 2 Materials and methods

### 2.1 Study area description

**Figure 1:**
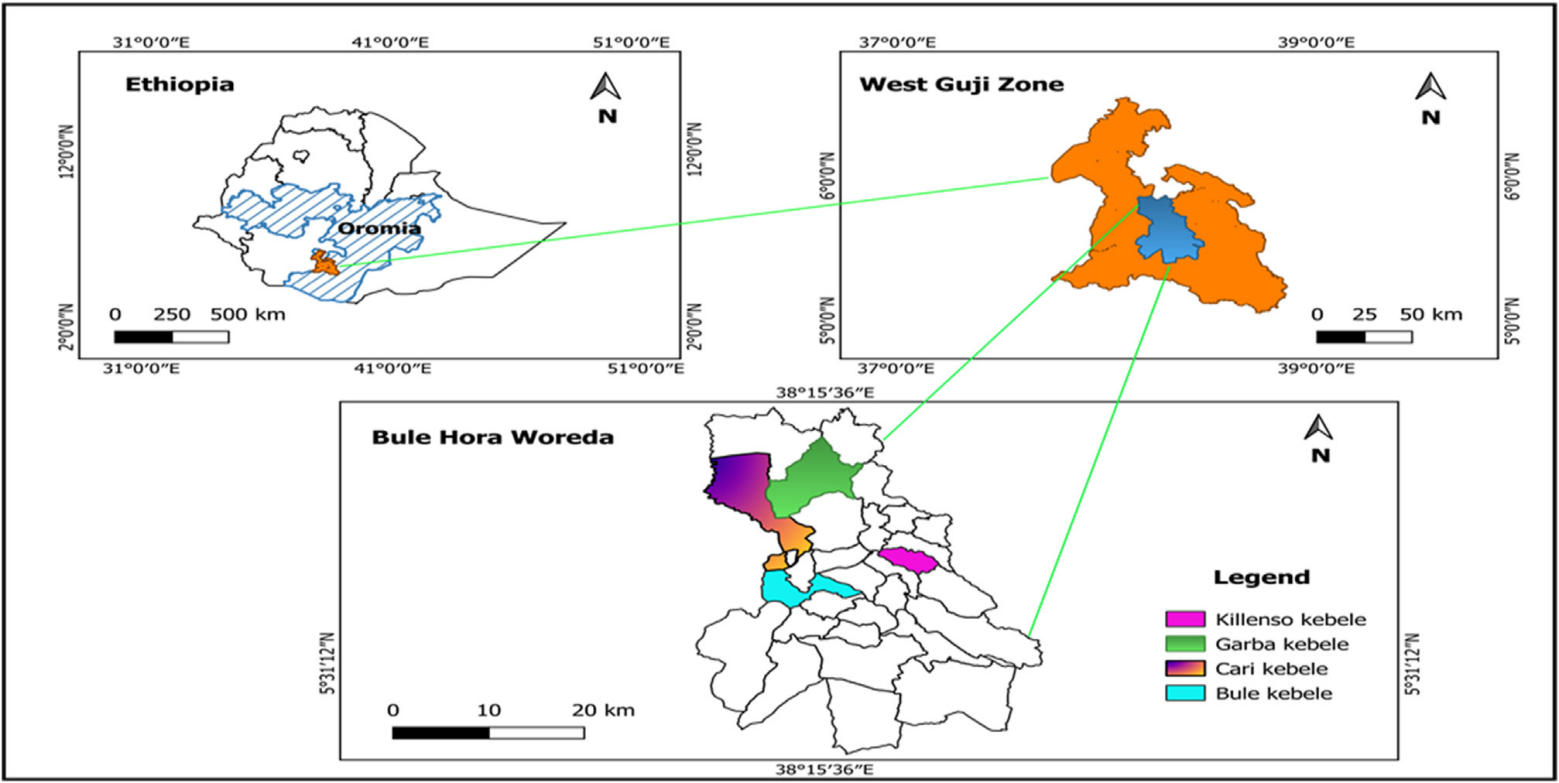
Location map of the Study sites.

This research was carried out in 2021 at four sites (*Kebeles*^1^) chosen from a total of forty *Kebeles* in the Bule Hora *Woreda*^2^ in the West Guji Zone of Southern Ethiopia (Figure 1). The area lies approximately 467 km from Addis Ababa, the capital of the country to the South. According to the Agricultural Office of the district (2020), the area lies between latitudes of 5º31′12″ North and longitudes of 38º15′36″ East. The district is divided into two agroecological zones: lowlands (≤1800 m a. s. l.), which account for 16.7% of the total land cover, and midlands (≤2500 m. a. s. l), accounting for 83.3 percent.

The area's rainfall pattern is bimodal, meaning that it has two different rainy seasons, with heavy rainfall between March and May and a reasonable amount from September to November. The temperature of the area is between 25 and 30 °C (Teshome, 2006).

Agriculture is the primary source of income, particularly for those living in rural areas. They grow a variety of both annual and perennial crops, including coffee (*Coffea arabica*), khat (*Catha edulis*), maize (*Zea mays*), wheat (*Triticum aestivum*), barley (*Hordeum vulgare*), teff (*Eragrostis tef*), bean (*Phaseolus vulgaris*), and enset (*Ensete ventricosum*) mainly in middle land areas. Another economic activity that directly supports farmers' economies is livestock production, which is primarily concentrated in lowland areas.

### 2.2 Sampling design and sample household determination

A reconnaissance survey was conducted to select sample sites. The sample study sites were chosen from two independent agroecological zones to compare farm-level crop diversity across altitudinal differences in the area (lower and mid-altitude areas). Following a previous study (Abebe, 2013), the distances of the locations from both the local market and the main road were also taken into account when selecting the sample sites, to investigate whether these factors influenced farmers' decisions on the growth of various crops. Farmers are encouraged to produce a range of crops because they can easily transport their agricultural products to customers owing to the proximity of markets and major highways. Due to the agro-ecological adaptability of crops to various environmental situations, the altitude of sites influences farmers' decisions to plant a variety of crops.

To examine crop diversification techniques among farmers of various wealth classes, sample households from each study site or kebeles were chosen depending on their wealth position. The total number of households in each study location was classified into three categories: poor, middle-income, and wealthy. The households were classified based on farm size and the number of cattle. When compared to those labeled as poor, individuals with significant farm sizes and cattle assets were considered wealthy. The poor have a tiny farm but no livestock, whereas middle-income households fall between the rich and the poor in terms of farm size and livestock numbers. Other characteristics of the sampled households were considered in this study, in addition to the financial level of the households (Table 1). The sample home determination process used by Getahun et al. (2016) was followed for each study location (kebeles). An equal number of sample households from each kebele was chosen to eliminate bias.

**Table 1:** List of explanatory variables used for model fitting and their specification with hypothesized effects on farm-level crop diversity.

Variables	Specification of Variables	Effects
Age	Age of sampled HH (year)	(+,-)
Farming experiences	HH farming experience (years)	(+,-)
Distance from market	the physical location of the areas from the local market (km)	(-)
Distance from Roads	the distances of the areas from the main roads (km)	(-)
Altitude	the altitudinal gradient of the studied sites (m. a.s.l)	(+)
Family Size	total family members of sampled HH	(+,-)
Farm Size	total farm size of the HH under cultivation of different crops (ha)	(+)
Livestock Assets	total number of livestock owned by sample HH	(+,-)
Income	Incomes of the HH year ^−1^ (ETB)	(+)
Education	∗educational level of sampled HH head	(+,-)
Wealth	∗wealth categories of the sampled HH	(+)

**Note**: HH = Households.

m.asl = Meters above sea level.

∗ Categorical predictor variables.

ETB = Ethiopian Birr.

A random sampling process was used to select sample households for each wealth class (poor, middle-income, and rich). We chose 84 household heads from four kebeles (21 household heads per kebele and 7 households from each wealth group), representing approximately 5% of the active farm households in each kebele. Because the local communities are semi-pastoralists, some of them have no permanent residence and live a nomadic lifestyle, traveling from place to place in search of grazing grounds for cattle. Even if they had permanent homes in some locations, their settlement pattern was dispersed rather than densely populated, making it difficult to sample many respondents. As a result, we limited the sample size for the analysis to 5% of the total households in each kebele.

### 2.3 Data collection

Data on socioeconomic and farm-level crop diversity were collected from four sites/kebeles in distinct agroecological zones between May and July 2021 (two in the lowlands and two in the midlands). Based on hypothesized criteria that influence crop diversification practices, Semi-structured questionnaires were developed**.** Socioeconomic data gathered using Semi-structured questionnaires included family size, educational level, farming experience, annual income, livestock size, and the distances of the sites from both the market and the main road. We pretested the questionnaire on five families from each site before the survey.

To estimate farm-level crop diversity, the farms were divided into two land-use types: home gardens (land used in and near a homestead) and crop fields (a plot further away from the homestead used to produce annual crops). Relevant crop diversity data were then gathered. To quantify farm-level agricultural diversity, the proportion of each crop species cultivated was assessed. Except for annual crops such as teff, maize, wheat, haricot bean, and barley, which are widely planted in vast agricultural areas, we assessed the area share of each crop species inside the home garden using meter tape.

Because of the large-scale farming of annual crops and the difficulties in measuring the area share of each crop species, the data were gained directly from the household survey. In a home garden with various inter-cropped crop species, the area share of the crops was determined by building plots within the home garden, particularly for perennials such as coffee and enset. Other sub-plots with dominant crop species were also built. When coffee and enset were inter-cropped and coffee was grown on a large-scale farm and enset was grown on a small-scale farm, a subplot with the dominant crop (coffee) was created within the main plots that included both coffee and enset and was specifically tailored for coffee. The area share of the dominant crop species (coffee) was subtracted from the total size of the main plot, and the area share of minor inter-cropped crop species (enset) was subtracted from the total size of the main plot.

The area share of root crops and vegetables grown on small-scale farms, primarily in home gardens, was calculated by measuring the area coverage of the species under cultivation using a meter tape. The area share under cultivation for fruit crops was estimated by measuring the canopy of the species, multiplying the individual number of species on the farm, and adding it all up.

### 2.4 Data analysis

Socioeconomic and on-farm data gathered using questionnaires were processed and organized for study. To assess farm-level crop diversity, three indices (Shannon-Wiener Index (SWI), species richness, and similarity coefficients) were adjusted using ecological measurements of spatial diversity in species (Magurran, 2004). SWI was calculated using the following equation:

$$H^{\prime }=\;\sum \mathrm{pilnpi}\;$$

Where; H′ **=** Shannon-Wiener diversity index and.

***Pi* =** proportion of area share of species i^th^ to the total area under cultivation.

This SWI was chosen because it produces a single index that combines richness and evenness (Wimp and Young, 2004). The similarity coefficients among the examined kebeles were calculated based on their crop species composition using Sorensen's (1948) equation, which is written as:

$$Ss=\frac{2Vij}{Vi+Vj}\;,$$

Where, **Ss** = Sorensen's similarity coefficient,
***Vij*** = the number of shared species between two regions and,
***V***_***i***_ and ***V***_***j***_ are the numbers of species in regions 1 and 2 respectively. The number of crop species grown in each household cultivation field was then counted. All of these farm-level crop diversity indices were calculated using Ecological Methodology version 7.4 software, which was created by Ecological Methodology for the analysis of ecological data (Krebs, 1999).

We examined the normal distribution and linearity of the data before starting data analysis to ensure that we met the multiple regression assumptions. A scatter plot was used to analyze the linear relationship between the dependent and predictor variables. The normal distribution of the data was further assessed using kernel density plots with a normal curve. Finally, various Stepwise Linear Regression (SLR) models were used to discover the most beneficial features of the crop diversity index. The dependent variable in this example was each crop diversity indices (species richness and Shannon-Wiener indices), whereas all other observed characteristics were treated as independent or predictor variables. Using SPSS statistical software tools, version 24, we analyzed the influence of socioeconomic variables on crop diversity.

## 3 Results

### 3.1 Diversity and composition of crop species in the studied sites

There were approximately 22 primary crop species found in both the lower and mid-altitude zones, with mid-altitude areas accounting for over 80% of the total and lowland areas accounting for the rest. Perennial crops, vegetables, and other root crops were cultivated in mid-altitude areas of home gardens, whereas households at lower altitudes mostly planted annual crops.

The cultivated area of each crop varied significantly across different Agro-ecologies (lower and mid-altitude) sites. Perennial crops such as coffee and enset are the most commonly cultivated crops in mid-altitude zones. Owing to their significant economic and consumptive value, households cultivate the two crops on comparatively large farms. Households cultivate enset for food all year, whereas households plant coffee primarily for profit. Wheat, maize, barley, and teff are among the other crops cultivated, but their cultivation area is tiny (less than 5 percent).

Compared to mid-altitude zones, lower altitude zones cultivate fewer crop species. Among the intensively grown crops, maize took the biggest farm size under cultivation (30%), possibly because of the crop's high consumptive value when compared to other crops, followed by wheat (25%), teff (20%), and haricot bean (20%). When compared to mid-latitude locales, coffee and enset farms have tiny farm sizes under cultivation (16% & 2 percent, respectively), suggesting that farm alterations in terms of altitudinal gradient not only affect the mix of crop species but also have a significant impact on crop distribution.

#### 3.1.1 Farm-level crop diversity indices

The farm-level survey used two crop diversity indices: species richness and Shannon-wiener indices. We counted the number of extant crop species on the farms to estimate species richness. The other farm-level crop diversity metric is the Shannon-Wiener index (SWI), which is calculated using the area proportion of crops under cultivation. We chose this indicator over the species richness index because it displays if a single species dominates a region.

Even though the number of crop species detected in Bule and Killenso kebele was identical, the Shannon-Wiener Index value in the two locations was not, as seen in the table (Table 2). Killenso kebele had a lower Shannon-Wiener index than the Bule kebele, showing that specific crops were more prevalent on larger farms. Cari kebele and Bule kebele both had more crop species (10) than Bule kebele (eight), however, Cari kebele had a lower SWI than Bule kebele. Garba kebele had a high SWI rating, showing that the existing crop species were sufficiently scattered across cultivated regions, as well as having a high species richness (21 species).

**Table 2:** Species Richness and Shannon-Wiener Index of the studied *Kebeles*

Diversity Indices	Agroecological Zone			
	Lowland *Kebeles*		Middle land *Kebeles*	
	Bule	Cari	Garba	Killenso
Species Richness	8	10	21	8
Shannon-Wiener Index	1.88	1.47	2.23	1.02

The average crop diversity index (SWI) among studied households was 1.66, with the majority (60 percent) having a diversity index of less than 1.5 (H′ 1.5), showing that farmers are not diversifying their crops. The remaining 40% of households had a high crop diversity score (H’ = 1.5–3.9), which could be attributed to a few farmers with large farms producing a variety of crops.

The Sorenson Similarity Index (SSI) was used to quantify the level of similarity among the study kebeles using the mix of crop species in each research site. 82% of the farmers in the lower altitude kebeles (Bule and Cari kebele) produced similar crop species (Table 3). When compared to lowland areas, mid-altitude kebeles (Garba kebele and Killenso kebele) had a low similarity index (59%) showing that crop diversification practices differed, most likely due to socioeconomic or physical factors influencing farmers' crop cultivation decisions.

**Table 3:** Sorenson Similarity Index (SSI) for the studied areas/*Kebeles* based on their crop species composition.

*Kebeles*	Bule	Cari	Garba	Killenso
Bule	1			
Cari	0.82	1		
Garba	0.39	0.48	1	
Killenso	0.53	0.56	0.59	1

The result of the Multiple Stepwise Linear Regression (SLR) analysis showed that altitude, distance from the main road and market, farm size, family size, number of livestock, yearly income, and wealth status influenced crop diversity (Table 4).

**Table 4:** Factors affecting farm-level crop diversity (n = 84).

Explanatory variables	Range of values	Shannon-Wiener Index	Crop species richness
		Standardized beta coefficient	Standardized beta coefficient
***Physical factor***			
Distance from the main road	0.02–17 km	-0.655∗∗	-0.715∗∗
Distance from the markets	0.5–9 km	-0.013	-0.271∗
Altitude	1706-2248 (m.a.s.l.)	0.311∗∗	0.626∗∗
***Socioeconomic factors***			
Farm size (ha)	1–10.25 ha	0.608∗∗	0.361∗∗
Number of Livestock	0-44 TLU	0.452∗∗	0.264∗
Income (ETB)	0-275,800 ETB	0.320∗∗	0.158
Family size	3-20 persons	0.303∗∗	0.282∗∗
Wealth Status		0.274∗	0.122
Age	21–90 years	-0.019	-0.066
Farming experiences	5–67 years	0.015	0.028
Education level	0–1 (0 - Unable to read & write; 1 – able to read & write)	0.058	0.098

**Note:** ∗, ∗∗, TLU: represent significance at P ≤ 0.05 and P ≤ 0.01, and (Tropical Livestock Unit) is a standard used to quantify different livestock types and sizes respectively.

## 4 Discussions

### 4.1 Effects of socioeconomic and physical factors on farm-level crop diversity

Crop diversity was negatively and significantly related to distance from the main road. Crop diversity indices at the farm level (Shannon-Wiener Index and crop species richness) decreased by 7% and 31% as the distance between the areas and the main road increased by one kilometer, while other factors remained constant. The effect was stronger in the second diversity measure (crop species richness) than in the first, showing that road inaccessibility has a greater impact on farm crop composition than on crop distribution. This means that farmers who live near all-weather roads grow a diverse range of crops. Garba, for example, was one of the study sites where a disproportionately large number of crop species were cultivated because of the main road that goes through it. In this area vegetables, fruits, and root crops, as well as perennial crops like enset and coffee widely cultivated. The area's proximity to the main road allowed farmers to transport and sell their farm products. This resulted in a diverse crop composition. In line with our findings, previous studies by Tobgay and McCullough (2008) and Mekuria and Mekonnen (2018) revealed that farmers who lived closer to highways were more likely to take part in markets and plant a crop mix than farmers who lived further away. Similarly, Faramarzy et al., 2019 found that the distance to a major road had a significant impact on diversity indices, however, the effects on agricultural variety were regional rather than global.

We found a positive and significant association between crop diversification and farm size. One of the major barriers to diversifying farmers' crops was the small amount of land they cultivated. Many small landowners have limited capacity to alter their crop farming because of their small farm size. This implies that as the agricultural area grows larger, farmers can plant different crops on each piece of land, contributing to farm-level crop diversification. Keeping all other variables constant, the farm-level crop diversity index (SWI) increased by 17% for every additional hectare of farmed land. This study supports Li et al. (2021), who found that increasing land size is a key element in boosting crop variety. Farmers with extensive landholdings would plant a variety of crops to suit their nutritional needs or sell surplus harvests to boost their income (Li et al., 2021). Many studies have showed a correlation between higher farm sizes and crop variety (Mekuria and Mekonnen, 2018; Faramarzy et al., 2019; Benin et al., 2004; Hosseinzadeh and Akhavan, 2015).

We found a positive relationship between crop diversification and livestock. The positive relationship between farm-level agricultural diversity and livestock size is because of the use of crop residue as feed, and the role of livestock in manure creation. According to the study, households with big livestock assets grew into a wider range of crops. This implies that the larger the herd, the more manure they produce. In contrast, crops such as enset and sugarcane provide nourishment for cattle, especially during droughts. It drew farmers with large animal flocks to diversify their crops. Similar to our finding, the study by Benin et al. (2004) confirmed that households with more livestock prefer to grow barley and wheat that produce fodder for livestock feeding.

Crop diversification has shown a positive relationship with the farm household's annual income. This implies farmers with higher annual incomes are more interested in crop diversification. According to Makate et al. (2016), farmers' income increased because of higher productivity through different cropping systems (crop rotations, inter-cropping).

Crop diversity and household size have a positive relationship. Because of multiple procedures, such as land preparation, sowing or planting crops, and harvesting, households with a large family size grew a larger range of crops. Previous studies (Benin et al., 2004; Aribi and Sghaier, 2020; Dembele et al., 2018; Abdalla et al., 2013; and Anuja et al., 2020) suggested that a big number of people in the farmers' families could add to the farm's variety of crops.

In this study, we found crop diversity inversely linked with market distance. Farmers that lived further distant from the market grew fewer crop species. It reduced crop species richness by 25% for every one-kilometer distance between the areas and the market, holding other factors constant. Similar to the findings of this study, Emana et al. (2015) found that poor transportation infrastructure and market access impeded smallholders’ adoption of high-value crops in Ethiopian rural areas. Other studies (McCullough and Pingali, 2008; and Kassie et al., 2011) reached similar conclusions, stating that the adoption of agricultural technology and high-value crop diversification was uncommon because of high transportation costs and a lack of market infrastructure.

Differences in farmer wealth have caused variation in farm-level crop diversification. Rich household farms had a minimum of one (1) SWI, whereas poor households' farms had a minimum farm-level crop diversity index (SWI) of 0.5, showing different crop varieties across wealth categories. Rich farmers may purchase basic agricultural inputs like fertilizer, a variety of seeds, and other supplies, allowing them to cultivate a wide range of crops. Similar to our finding, Gentle and Maraseni (2012) found that poor households had fewer opportunities to diversify because of limited land ownership, whereas wealthy households could introduce new crop types because of sufficient farm size for the species’ production.

Crop diversification levels in the studied locations varied significantly across altitudinal changes. Because the places shared climatic characteristics from both lower and higher altitude zones, various crop species adapted well to mid-altitude agro-ecological environments, allowing farmers to grow a variety of crops. Despite the notion that mid-altitude areas are expected to have a wide range of crop species, the study found that some mid-altitude locations had restricted crop diversity, which was most likely owing to socioeconomic constraints that limit farmers' options to plant a diverse crop mix.

Crop diversity was unaffected by socioeconomic factors such as the age and agricultural experience of the households, as well as their educational levels, in the current study. The age of the families, however, is inversely associated with farm-level crop diversity, showing that as people get older, their participation in agricultural activities reduces, affecting farm-level crop diversity.

## 5 Conclusion recommendations

Crop diversity is critical on the farm, especially for small-scale farmers who rely on it for a living. Crop diversification strategies differed among farms because of the farmers' socioeconomic considerations and the crop species' adaptability to unique environmental circumstances. In terms of road and market accessibility, inaccessibility has a negative and considerable impact on farmers' crop diversification decisions. Farmers who were far from the road and the market produced fewer specific crop species than those who were near both. Crop diversity has positively associated with the size of a household's farm and animals, annual agricultural product income, and the total number of family members. Because of the huge farm sizes, it actively encouraged farmers to produce a range of crops. We also found the altitudinal gradient of the land positively associated with the composition and distribution of crops. Farms in the mid-latitudes had perennial crops and some vegetables all year, whereas farms at lower elevations had only a few seasonal crop species, annual crops.

Based on the findings of the study, the practices of farm-level crop diversification can be boosted through:

❖ Creating awareness for farmers, particularly those living in lower altitude areas, who cultivate less diversified crops.
❖ Creating market links for agricultural products and improving road accessibility.
❖ Because this study concentrated on socioeconomic issues, more research is needed to examine the impact of environmental factors such as soil characteristics and temperature variability across different agro-ecological zones on farm-level crop diversification.

## Declarations

### Author contribution statement

Belay Maru; Melesse Maryo; Getahun Kassa: Analyzed and interpreted the data; Wrote the paper.

### Funding statement

This research did not receive any specific grant from funding agencies in the public, commercial, or not-for-profit sectors.

### Data availability statement

Data will be made available on request.

### Declaration of interests statement

The authors declare no conflict of interest.

### Additional information

No additional information is available for this paper.

## Footnotes

1 *Kebele* is the lowest administrative unit in Ethiopia usually inhabiting more than 300 households.

2 *Woreda* is the third level of the administrative division of Ethiopia after zones and the regional states.
